# A population-based study of pain and quality of life during the year before death in men with prostate cancer

**DOI:** 10.1038/sj.bjc.6601654

**Published:** 2004-02-24

**Authors:** G Sandblom, P Carlsson, K Sennfält, E Varenhorst

**Affiliations:** 1 Department of Surgery, Uppsala University Hospital, 751 85 Uppsala, Sweden; 2 Centre for Assessment of Medical Technology, Linköping University, 581 85 Linköping, Sweden; 3 Centre for Assessment of Medical Technology, Linköping University, 581 85 Linköping, Sweden; 4 Department of Urology, Faculty of Health Sciences, Linköping University, 581 85 Linköping, Sweden

**Keywords:** prostate cancer, quality of life, pain, terminally ill

## Abstract

In order to explore how health-related quality of life changes towards the end of life, a questionnaire including the EuroQOl form and the Brief Pain Inventory form was sent to all men with prostate cancer in the county of Östergötland, Sweden, in September 1999. Responders who had died prior to 1 January 2001 were later identified retrospectively. Of the 1442 men who received the questionnaire, 1243 responded (86.2%). In the group of responders, 167 had died within the study period, 66 of prostate cancer. In multivariate analysis, pain as well as death within the period of study were found to predict decreased quality of life significantly. Of those who died of prostate cancer, 29.0% had rated their worst pain the previous week as severe. The same figure for those still alive was 10.5%. On a visual analogue scale (range 0–100), the mean rating of quality of life for those who subsequently died of prostate cancer was 54.0 (95% confidence interval ±5.2) and those still alive was 70.0 (±1.2). In conclusion, health-related quality of life gradually declines during the last year of life in men with prostate cancer. This decline may partly be avoided by an optimised pain management.

In recent years, health-related quality of life has become one of the most important end points for studies on men with prostate cancer, especially men with cancer at an advanced stage. Caring for cancer patients in the last years of life requires a good understanding of how health-related quality of life can be improved when efforts to prolong life become increasingly futile. Adequate pain and symptom management, avoidance of inappropriate prolongation of the dying process, achievement of a sense of control, relief of burdens, and the strengthening of relations with loved ones have been found to be the most important domains from the patient's perspective of palliative treatment (Singer *et al*, 1999). Of all factors affecting health-related quality of life, however, pain is usually considered the most prominent factor for patients approaching death (Elliott, 1997).

Although pain management is fundamental in terminal care (Steinhauser *et al*, 2000; Wrede-Seaman, 2001), it is often undertreated (Perron and Schonwetter, 2001). According to the World Health Organisation's guidelines for the stepwise management of pain in cancer, the potency of the analgesia provided, ranging from non-opioids and opioids for moderate pain (e.g. dextropropoxyphene, codeine and tramadol) to opioids for severe pain (e.g. morphine, cetobemidone and fentanyl), should be adjusted to the patient's experienced level of pain (WHO, 1986). If this is implemented consistently and proper palliative approaches are maximised, it has been claimed that effective pain control may be achieved in 80% of cancer patients (Perron and Schonwetter, 2001). However, despite the widespread acceptance of this strategy, more than 40% of prostate cancer patients in routine practice settings report the presence of pain (Greenwald *et al*, 1987; Portenoy, 1989; Larue *et al*, 1995; Sandblom *et al*, 2001).

Health-related quality of life in the final year of life in terminally ill patients, including men with prostate cancer, has been studied previously (Liao *et al*, 2000; Litwin *et al*, 2001; Melmed *et al*, 2002). However, in order to reach a full understanding of how health-related quality of life is affected by approaching death, all patients in a population-based sample must be studied. We have therefore analysed the outcome of a questionnaire that was sent to all men with prostate cancer in the county of Östergötland, Sweden, in 1999. The questionnaire consisted of EuroQol, designed to evaluate health-related quality of life, and Brief Pain Inventory Form (BPI), which consists of questions with regard to the severity and impact of pain on daily functions. The results from this study have been published previously (Sandblom *et al*, 2001). We now present a separate analysis of men in the same cohort who died before 1 January 2001, who were identified by crosslinking with the National Death Register.

## MATERIAL AND METHODS

### Study base

The basic source of information was a questionnaire sent to all men with prostate cancer in the county of Östergötland. Östergötland is one of three counties of the South-East Health Care Region of Sweden (Östergötland, Jönköping and Kalmar county). The total population in Östergötland in 1999 was 412 000. It has two peripheral hospitals and one central referral hospital. All cases of prostate cancer were identified in the National Tumour Register, which was started in 1958 as a population-based cancer register and has a coverage greater than 98% (Mattsson, 1977). It contains data on all tumours diagnosed, including personal number and date of diagnosis. For cases diagnosed in 1987 or later additional data on tumour stage, grade and treatment were extracted from the South-East Region Prostate Cancer Register, which serves as an extension of the National Tumour Register (Sandblom *et al*, 2000). The register has been validated and shown to have a high reproducibility (Sandblom *et al*, 2003). For cases diagnosed prior to 1987, data were achieved through a review of case histories at each respective urology department. The central death register was searched for cases who had died before the start of the study period and these were excluded. All data from the questionnaire were converted into electronic form by scanning and then checked once manually.

In 2003, the central death register was checked for men included in the study who had died within 1 year of the day the questionnaire was distributed. In this study, we primarily used the cause of death recorded in the South-East Region Prostate Cancer Register. When cause of death was not registered in the South-East Region Prostate Cancer Register, we relied on the cause of death registered in the central death register.

### Subjects

There were 7199 cases of prostate cancer diagnosed in Östergötland registered in the National Tumour Register up to 31 December 1998; of these 4474 were registered as dead in the Tumour Register. In all, 30 cases were excluded due to incomplete personal registration number, diagnosis before 1969, or because they were born before 1900. The remaining 2695 were crosslinked with the National Population Register, which resulted in the further exclusion of 1145 deceased men, eight men, where matching with the personal registration number was impossible to achieve, and 40 men who had left the county. In a repeat match with the population register in November 1999, an additional 60 deaths were discovered, leaving 1442 cases in the group studied.

The first letter with the questionnaire and an explanation was sent in September 1999. Two further letters were sent 2 and 4 weeks after the first letter as reminders to nonresponders. A nurse was available on the telephone at each of the three urology departments in the county for general information and clarification of the questions.

### Questionnaire

The questionnaire was a combination of the EuroQol, parts of the BPI and eight specially designed questions. Altogether, there were 26 questions.

EuroQol is a nondisease-specific instrument for describing and evaluating health-related quality of life (EuroQol© Group, 1990). It was developed as an internationally standardised complement to other health status measures, having five questions covering the basic domains common to generic health status and a visual analogue scale (VAS) for the indication of general health state. The answers to the first five questions can be derived to produce an overall index of health status (EQ-5D). A validation of EuroQol in Sweden has shown a striking similarity to results from other European centres (Brooks *et al*, 1991).

The BPI is an instrument designed to assess the severity of pain and impact of pain on daily functions among patients with cancer pain and pain due to chronic disease. It rates the degree to which pain interferes with mood, walking and other physical activities, work, social activity, relationships with others and sleep. The BPI has been validated in several studies (Serlin *et al*, 1995; Cleeland *et al*, 1996). In our study, we included the four BPI questions related to pain intensity (pain now, worst pain last weak, least pain last weak and average pain last week) and the seven questions related to pain interference with daily functions (interference with general activity, mood, walking, work, relations with others, sleep and enjoyment of life).

In addition to the standardised questions from EuroQOL and BPI, eight specially designed questions inquiring about the effectiveness of pain treatment, whether pain treatment was given in time, side effects of treatment, which medications had been prescribed, civil state and how easy it was to get in contact with a nurse or doctor when needed were also included.

### Pain management index

To determine whether the patient was adequately managed for his pain, a pain management index (PMI) was determined (Cleeland *et al*, 1994). The index was derived by subtracting the rating of worst pain on the BPI questionnaire from a score corresponding to the strongest prescribed analgesic as reported by the respondent. The analgesic drug score was defined according to the WHO's analgesic ladder: 0 for no analgesic, 1 for nonopioids, 2 for opioids for moderate pain, and 3 for opioids for severe pain. Based on the worst pain as stated in the BPI questionnaire, the pain score (0–10) was categorised as 0 for no pain (rating 0), 1 for mild pain (rating 1–3), 2 for moderate pain (rating 4–7) and 3 for severe pain (rating 8–10). A negative score indicates undertreatment of the pain (Cleeland *et al*, 1994). Although PMI is not accurate for prescribing drug to an individual, it provides a rough estimate of how pain is treated in the population.

### Statistics

In the analyses, localised tumours were defined as T0-2, NX/N0 and M0, and all others were treated as advanced. The treatment was categorised into three groups: watchful waiting; palliative treatment (including bilateral orchiectomy, GnRH-analogues, transurethral resection of the prostate, antiandrogens and oestrogen); and treatment with curative intent (including radical prostatectomy, external radiation therapy and brachytherapy). In a multivariate regression analysis, factors predicting outcome from the BPI question with regard to ‘worst pain the last week’ (11 grades) were assessed, including patient age, civil state, time since diagnosis, presence of distant metastases at time of diagnosis, last received treatment, rating of health-care availability and death within 1 year of questionnaire distribution as independent variables. Treatment was divided into palliative, curative and watchful waiting, with watchful waiting considered as reference. The rating of health care availability was divided into three categories: ‘no need of contact’; ‘easy to get in contact’ (always easy or usually easy to get in contact); and ‘difficult to get in contact’ (neither easy nor difficult, usually difficult and always difficult). ‘No need for contact’ and ‘difficult to get in contact’ were included in the analysis and ‘easy to get in contact’ was treated as reference. Death within 1 year of distribution of the questionnaire was divided between death from prostate cancer and death from other causes.

Similarly, factors predicting health-related quality of life as stated on the VAS in the EuroQol questionnaire were assessed in a multivariate regression analysis, with age, civil state, time since diagnosis, tumour stage (localised/advanced), last received treatment, the rating of ‘pain on average the last week’, rating of health-care availability and death within 1 year of questionnaire distribution, as independent variables. A multivariate logistic regression analysis with age, treatment, civil state, presence of distant metastases at diagnosis, time since diagnosis, rating of health-care availability and death within 1 year of questionnaire distribution was used to assess risk factors for negative PMI.

## RESULTS

Of the 1442 who received the questionnaire, 1243 (86%) responded (Table 1

**Table 1:** Distribution of age, ratings of quality of life and number of patients taking strong opioids for patients who died of prostate cancer, patients who died of other causes and patients still alive (*n*=1242, cause of death not registered for one patient)

	**Died of prostate cancer**	**Died of other causes**	**Still alive 31 December 2000**
Number	66	100	1076
Age (years, ±s.d.)	76±10	82±6	77±8
Eq5D score (±95% confidence interval)	0.538±0.077	0.564±0.067	0.770±0.015
EuroQOL VAS (±95% confidence interval)	54.0±5.2	53.2±4.6	70.0±1.2
Number of patients receiving strong opioids	17 (25.8%)	3 (3.0%)	15 (1.4%)

VAS=visual analogue scale.

). The reasons for drop out were: absence of response (*n*=145); inability to answer due to disease (*n*=34); absence of answers in the returned questionnaires (*n*=8); refusal to answer (*n*=5); not reachable at noted address (*n*=4); change of address (*n*=1); answered by wrong person (*n*=1); and too incoherent answers to allow adequate interpretation (*n*=1). Of the 1243 responders, 78 had prostate cancer diagnosed before 1987.

In the group of responders, 167 (13.4%) died before 1 January2001, 66 (5.3%) of prostate cancer and 100 (8.0%) of other causes. Cause of death could not be identified for one patient. Of the 199 nonresponders, 70 (35.2%) had died before 1 January 2001, 20 (10.1%) of prostate cancer. From now, all analyses refer to the 1243 responders.

The primary treatment of the 1243 responders was distributed between watchful waiting (*n*=582), palliative treatment, including bilateral orchiectomy (*n*=127), GnRH analogues (*n*=238), transurethral resection of the prostate (TUR-p, *n*=37), antiandrogens (*n*=15), oestrogen (*n*=8), and treatment with curative intent, including radical prostatectomy (*n*=156), external radiation therapy (*n*=58) and brachytherapy (*n*=16). Information on treatment was missing for six cases. Of those initially managed with watchful waiting, 15 later received treatment with curative intent and 184 received palliative treatment. Similarly, of those who initially were treated with curative intent, 30 patients later received palliative treatment, and of those initially receiving palliative treatment four were later treated with curative intent. At the time of the questionnaire, 383 men were thus managed with watchful waiting, 635 received palliative treatment and for 219 treatment with curative intent was registered as the last treatment received.

In multivariate regression analyses, death before 1 January 2001 was found to be a significant factor predicting the rating of ‘Worst pain last week’ in the BPI as well as health-related quality of life as estimated by the VAS in the BPI (Tables 2

**Table 2:** Variables predicting ‘worst pain last week’ in a multivariate regression analysis

	***t*-value**	**Significance**
Health-care availability	5.32	<0.001
Dead before 31 December 2000	3.90	<0.001
Time since diagnosis	1.88	0.061
Treatment with curative intent	−1.66	0.097
Civil state	1.29	0.20
Palliative treatment	1.11	0.27
Age (years)	−0.36	0.72
Presence of distant metastases at diagnosis	0.08	0.94

and 3

**Table 3:** Factors predicting health-related quality of life as estimated on the EuroQol VAS in a multivariate regression analysis

	***t*-value**	**Significance**
Worst pain last week	−15.47	<0.001
Dead before 31 December 2000	−5.91	<0.001
Age (years)	−5.84	<0.001
Health-care availability	−4.90	<0.001
Palliative treatment	−2.26	0.024
Time since diagnosis	−0.71	0.48
Tumour stage at diagnosis	0.50	0.62
Civil stage	0.26	0.80
Treatment with curative intent	−0.043	0.97

VAS=visual analogue scale.

, Figures 1

**Figure 1:**
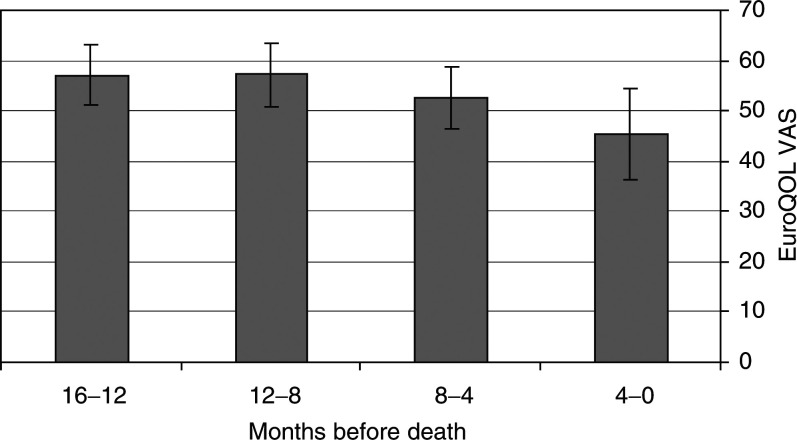
Quality of life, as rated on the EuroQol VAS, during the last 16 months of life. ± 95% confidence interval.

, 2

**Figure 2:**
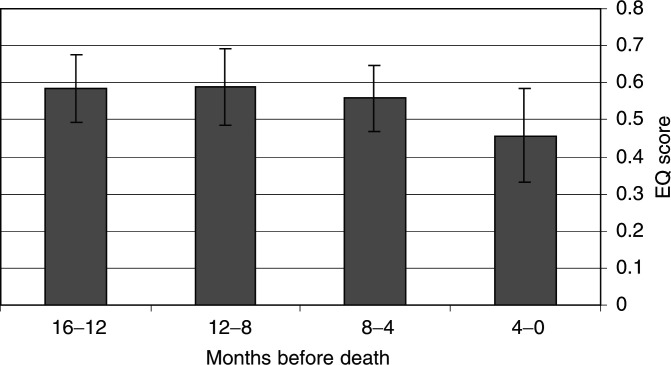
Quality of life, as estimated by the EQ-5D score, during the last 16 months of life. ± 95% confidence interval.

and 3

**Figure 3:**
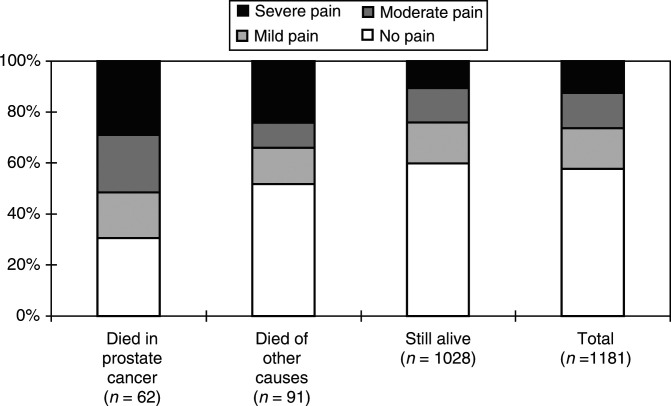
Distribution of ratings of ‘worst pain last week’ in BPI (number of responders within parenthesis).

). For PMI, however, death during the period of study was not found to be a significant predictive factor in a multivariate logistic analysis (Table 4

**Table 4:** Factors predicting a negative pain management index in a multivariate logistic analysis

**Factor**	**Estimated odds ratio**	**Significance**
Health-care availability	0.69	<0.001
Distant metastases at diagnosis	2.89	0.001
Treatment with curative intent	0.66	0.053
Dead before 31 December 2000	1.13	0.538
Civil state	1.10	0.560
Time since diagnosis	1.00	0.576
Palliative treatment	0.98	0.914
Age (years)	1.00	0.992

).

Men who died of prostate cancer were found to report ‘worst pain last week’ significantly higher than men who died of other causes when testing with Mann–Whitney *U*-test (*P*<0.05). There were only minor nonsignificant differences in health-related quality of life between those who died of prostate cancer and those who died of other causes (Table 1).

**Figure 4:**
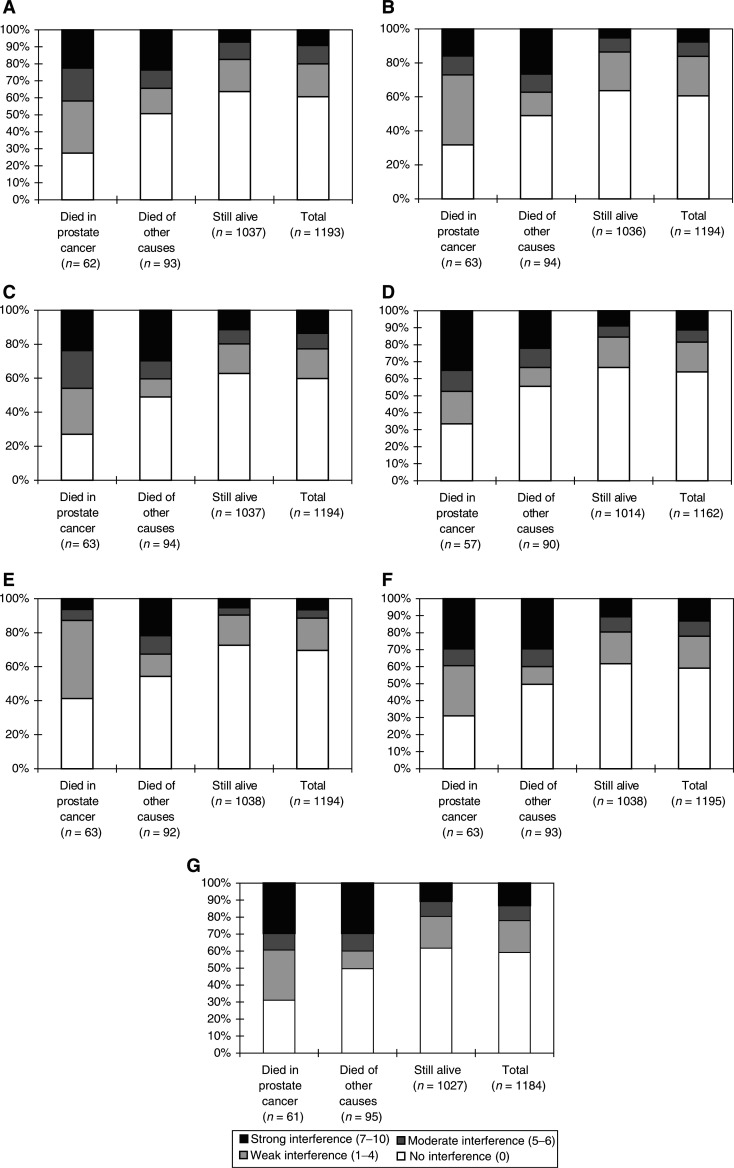
(**A**) Distribution of answers to the BPI question of how pain interferes with general activities (number of responders within parenthesis). (**B**) Distribution of answers to the BPI question of how pain interferes with mood (number of responders in brackets). (**C**) Distribution of answers to the BPI question of how pain interferes with walking (number of responders within parenthesis). (**D**) Distribution of answers to the BPI question of how pain interferes with work (number of responders within parenthesis). (**E**) Distribution of answers to the BPI question of how pain interferes with relations with other people (number of responders within parenthesis). (**F**) Distribution of answers to the BPI question of how pain interferes with sleep (number of responders within parenthesis). (**G**) Distribution of answers to the BPI question of how pain interferes with enjoyment of life (number of responders within parenthesis).

For all BPI questions with regard to interference of pain with daily functions, a significant difference in the ratings were seen between those who died within the study period and those still alive (all *P*<0.005). Except for interference with work (*P*<0.05), no significant difference was seen between those who died of prostate cancer and those who died of other causes for any of the pain interference questions (Figure 4A–G

)

## DISCUSSION

The present study represents the outcome of a geographically defined cohort of men with prostate cancer in their final year of life. The population-based design helps to avoid bias due to selection of patients managed at specialised units and other selection processes associated with the disease or general health status of the patient. Several of the previously published studies of health-related quality of life in the final years of life are based on patients treated at referral centres, which inevitably results in a convenience sample. A minor source of bias in the present study are the nonresponders, who were over-represented among those who died within 1 year of questionnaire distribution. In this group, there may be a number of patients who were unable to answer the questionnaire since they had reached the terminal stage of their disease.

Of those who died of prostate cancer, 27.6% stated their ‘worst pain last week’ as severe. Whether this should be considered acceptable and reflecting optimal pain treatment is an open question. With the updated interpretation of the WHO analgesic ladder (Coluzzi, 1998), which advocates earlier introduction of opioids, unnecessary suffering from chronic malignant pain may in many cases be avoided. It has been claimed that it is possible to achieve good pain relief in 90–95% of dying patients (Elliott, 1997; Harrold, 1998). However, the reasons for pain in men with advanced prostate cancer are often complex and may need approaches other than opioid treatment. Many of these patients probably suffer not only from nociceptive pain but also from neuropathic pain due to metastases compressing nerve rotes. This type of pain is not completely opioid responsive (Arner and Meyerson, 1988; Cherny and Portenoy, 1994) and may require surgical neurectomy or anaesthetic block techniques. Patients with metastases to the retroperitoneal lymph nodes or the liver may also experience visceral pain. Intolerance to opioid treatment is a further problem that should not be ignored. If side effects of the treatment, such as constipation and nausea, are not managed carefully, the reduction of health-related quality caused by the disease itself may be even further pronounced. Despite the effectiveness of opioids for treatment of nociceptive pain, complete pain relief is not always achievable. The complex nature of cancer pain makes it unlikely to reach a complete pain relief for more than 90% of these patients, even under optimised circumstances (Serlin *et al*, 1995; Zech *et al*, 1995).

In the case of widespread skeletal metastases, radiotherapy directed against painful bone metastases or intravenous treatment with radionuclides relieves pain effectively. Bisphosphonates or corticosteroids may also be used as adjuvant pain treatment to men with painful bone metastases. In addition to pharmacological pain treatment, there are a number of alternative nonpharmaceutical approaches that have not been fully evaluated, such as transcutaneous electrical nerve stimulation, acupuncture or massage therapy. Although the mechanisms for these types of treatment are poorly understood, they can be attempted as a complement to pharmacological treatment.

Since no palliative therapy other than hormonal treatment has been consistently recorded in the South-East Region Prostate Cancer Register, we do not know how patients with hormone-refractory prostate cancer have been treated. Radiotherapy and radionuclide treatment are established as standard in the South-East Region for treating men with painful metastases and has probably been given to the majority of these patients. All other treatments have been provided on individualised terms.

Despite the absence of a significant association between death within the period of study and negative PMI, there may still be a number of undertreated patients. Difficulties in getting into contact with the health-care facilities, dissimulation or inappropriate reluctance to take strong opioids for fear of addiction or side effects may result in patients not receiving the treatment they require. By constructing a model based on the same cohort as in the present study, it was shown that 0.82 quality-adjusted life-years could theoretically be added to every man in the cohort if pain treatment is optimised (Sennfält *et al*, 2003).

Since we do not know how many of the men were still working when they answered the questionnaire, it is difficult to interpret the question with regard to the impact of pain on work. There were 98 men (7.9%) who were 65 years or younger, which is the age when you are retired in Sweden if you do not receive early retirement pension. However, it is difficult to give any other explanation for the difference between men who died of prostate cancer and those who died of other causes probably than that men in the first group were more disabled by their disease (Figure 4D).

A decrease in health-related quality of life was seen during the final year of life, especially during the final 4 months (Figure 2). Designing a questionnaire that reflects all the physical, psychological, social and spiritual changes that take place towards the end of life, each interfering with all the others, would be extremely difficult. However, the VAS as well as the EuroQol index give a good overall depiction of how health-related quality of life is affected. Only the patient himself can truly define his own quality of life, which is the strength of the VAS.
